# Supplementary material for: Menstrual changes after COVID-19 vaccination among menstruators of reproductive age: A cross- sectional study from Erbil City, Iraq
Source: PLOS Glob Public Health. 2024 Apr 4;4(4):e0003079. doi: 10.1371/journal.pgph.0003079 (PMC10994343; doi:10.1371/journal.pgph.0003079)
Supplement: S1 File — (PDF) [file pgph.0003079.s001.pdf]

Questionnaire: Menstrual Changes after COVID-19 Vaccination among Menstruator of Reproductive Age: A Cross- Sectional Study from Erbil City, Iraq

Code ………………. Date………………………

I- Demographic data

1-Age …………….

2- Educational level:

Illiterate Primary Secondary Institute College High degree

3- Occupational:

Formal employment Self-employed Unemployed Student

4-Maternal status: Married Unmarried

5- Menstrual age

II- Information regarding Covid-19 Vaccination and Menstrual cycle.

Have you contaminated by coved 19? 1-Yes 2- No

1- Have you received coved 19 vaccine?

If yes, answer the from questions (2 to end). If no, answer from questions (3 to7)

2-Which types ……………………….

Who many dosages you are received: 1st 2nd 3rd

Date of 1st dosage ……………….............

Date of 2nd dosage ………………………..

Date of 3rd dosage ………………………….

Before contaminated by Coved 19 and received Coved 19 vaccine

3-Are your periods regular? 1- yes 0- no

4-How many days of bleeding do you usually have each period? …………………..

5-How heavy is your menstrual flow usually?

 Light

 Moderate

 Heavy (clots/flooding)

 Can’t remember

6-How many days are there between the start of one period and the start of the next on average?

 Less than 21 days

 22-24 days

25-28 days

 29-32 days

33 – 35 days

 More than 36 days

Too irregular to say

7- Have you had any of these symptoms before received coved 19 vaccine?
Menstrual pain? (dysmenorrhea)

Lower back pain

Nausea

Genital rash

Vaginal irritation

Genital redness or inflammation

Questions a about periods after received coved 19 vaccine or contaminated by Coved 19

8-Are your periods regular after received coved 19 vaccine? 1- yes 0- no

9-How many days of bleeding do you have a after received coved 19 vaccine?............

10-How heavy is your menstrual flow after received coved 19 vaccine?

 Light

 Moderate

 Heavy (clots/flooding)

 Can't remember

11-How many days are there between the start of one period and the start of the next on average after received coved 19 vaccine?

 Less than 21 days

 22-24 days

25-28 days

29-32 days

33 – 35 days

More than 36 days

Too irregular to say

12- Have you had any of these symptoms after received coved 19 vaccine?

Menstrual pain? (dysmenorrhea)	1- yes	0- no
Lower back pain	1- yes	0- no
Nausea	1- yes	0- no
Genital rash	1- yes	0- no
Vaginal irritation	1- yes	0- no
Genital redness or inflammation	1- yes	0- no

Time of the period return to normal like pre-contaminated by Coved 19 ……………….

Time of the return to normal like pre- received coved 19 vaccine……………………….
