# Supplementary material for: Menstrual changes after COVID-19 vaccination among menstruators of reproductive age: A cross- sectional study from Erbil City, Iraq
Source: PLOS Glob Public Health. 2024 Apr 4;4(4):e0003079. doi: 10.1371/journal.pgph.0003079 (PMC10994343; doi:10.1371/journal.pgph.0003079)
Supplement: Attachment — Submitted filename: Response to Reviewers.docx [file pgph.0003079.s002.docx]

**Manuscript: PGPH-D-23-00059**

**Response to Reviewers**

**Dear Dr. Jianhong Zhou**

Thank you very much for giving Me the opportunity to revise and resubmit the manuscript entitled ‘‘Menstrual Changes after COVID-19 Vaccination among Women of Reproductive Age: A Cross- Sectional Study from Erbil City, Iraq’’. I deeply appreciate the time and effort that the academic editor and the reviewers devoted to provide comments and suggestions on my manuscript. I’m is grateful for their invaluable comments as they enriched and improved the manuscript.

I have carefully considered and responded to each comment made by the editor and the reviewers and I have successfully incorporated most of the suggestions into the revised manuscript.

The corresponding changes are highlighted “yellow” in the manuscript text in the revised file.

Thank you again for your consideration of my revised manuscript.

Sincerely,

Dr. Warda Hassan Abdullah

**Editor Comments**

**Comment 1:** In the ethics statement in the Methods, you have specified that verbal consent was obtained. Please provide additional details regarding how this consent was documented and witnessed, and state whether this was approved by the IRB.

**Author response:** I have made several additions to the manuscript. First, I have included detailed information regarding participant consent in the ethics statement. Furthermore, I obtained approval from the ethical committee of the College of Nursing, Hawler Medical University, which granted permission to initiate the study. It is important to note that we do not have an Institutional Review Board (IRB), but we do have an ethical committee at our College and University to oversee ethical considerations. The approval from this committee ensures that the study adheres to ethical guidelines and safeguards the rights and well-being of the participants.

In the present study, verbal informed consent was obtained from all the menstruators who participated. This consent process took place during the initial gathering at the primary health care centers, where the researcher explained the study's purpose and details to the participants and invited them to provide informed consent verbally. Verbal consent was chosen as the preferred method due to its greater acceptance in our society, as individuals generally exhibit hesitancy towards giving written consent.

I have made revisions of the ethics approval and consent to participate section to provide more detailed information about the verbal consent.

**Comment 2:** We ask that a manuscript source file is provided at Revision. Please upload your manuscript file as a .doc, .docx, .rtf or .tex.

**Author response:** I want to assure you that I have uploaded the manuscript file in the appropriate formats. The file has been submitted as a .doc, .docx, .rtf, or .tex file during the revision process.

**Comment 3:** We have noticed that you have uploaded Supporting Information files, but you have not included a list of legends. Please add a full list of legends for your Supporting Information files after the references list.

**Author response:** I want to confirm that when re-submitting the revised manuscript, I made sure to include a comprehensive list of legends for all Supporting Information files in the manuscript.

**Comment 4:** Please amend your Data Availability Statement and indicate where the data may be found.

**Author response:** I make sure that upon re-submitting the revised manuscript, I upload the study’s minimal underlying data set as Supporting Information files.

**Additional Editor Comments:**

Dear Editor,

I am writing to inform you that I have thoroughly revised the manuscript in general:

**Introduction and Justification:** I have reworked the introduction to clearly state the research objectives and address the existing literature on the topic. I have provided a more comprehensive justification for the study, highlighting the research gap that my work aims to fill.

**Methods:** I have carefully reviewed the methods section and made necessary clarifications to address the concerns raised by the reviewers. I have provided additional details regarding the survey instrument creation, including its validation and reliability measures.

**Results:** I have reorganized and presented the results in a more logical and coherent manner. I have ensured that the key findings are accurately and clearly reported, addressing the objectives of the study.

**Discussion:** I have thoroughly reworked the discussion section to improve its coherence and logical flow. I have integrated the relevant literature more effectively, highlighting the implications of our findings and their contribution to the field. I have also addressed the limitations of the study and provided suggestions for future research directions.

**Tables:** I have carefully reviewed and revised all tables, ensuring their clarity and alignment with the revised content.

**References: Adding and update**

Thank you for your time and consideration.

Sincerely,

**Reviewers Comments**

**Response to Reviewer: 1**

**Reviewer 1: General Comments:**

**Comment 1:** With recognition of those with gender-nonconforming identities such as transgender men and some non-binary persons using the term women to indicate those that menstruate is not inclusive. Please instead use the term ‘menstruator’ of reproductive age. This could even be turned into an acronym MORA. In the new definition for Menstrual Health this term is used along with a justification of using gender inclusive terms if you’d like a reference <https://www.tandfonline.com/doi/full/10.1080/26410397.2021.1911618>

**Author response:** Thank you for your feedback and valuable suggestions regarding my manuscript. I have made a change throughout the entire manuscript, replacing the term "Women" with "Menstruator of Reproductive Age (MORA)," and I have also introduced it as an acronym, MORA.

**Comment 2:** The English is not perfect throughout the document – I’m not sure how much this matters as I can still mostly understand the meaning, but other non-native English speakers may struggle. It would take too long to list every error – perhaps just give it another read through, or ask a colleague to check it? A general edit to make is that you often repeat the same word in one sentence unnecessarily e.g., women or disease in the introduction. I’m unsure if this journal offers proofreading for non-native English writers?

**Author response:** Thank you for your feedback and valuable suggestions regarding the manuscript. I appreciate your understanding of the overall meaning, despite the imperfections in the English language usage.

**I have sent the manuscript to an English language expert for proofreading and correction. Additionally, I have rewritten the manuscript based on their feedback.**

Comment 3: A strong narrative would be to point out that women’s health is often neglected from data collection, which is why they are often misdiagnosed or not diagnosed at all – symptoms of autism and heart attacks are based on the male experience. Ref – book, Invisbile Women, Caroline Criado Perez. So I imagine that this was the case for testing the COVID vaccine too – that and the invisibility of menstrual health. I think this would strengthen the paper.

**Author response:** Thank you for your comment. I appreciate your perspective on the importance of considering gender bias in data collection and the potential impact it can have on misdiagnoses and underdiagnoses, as highlighted in Caroline Criado Perez's book, 'Invisible Women.' I agree that this issue is significant and relevant not only in women's health but also in various aspects of medical research, including the development and testing of the COVID vaccine.

I believe that discussing the invisibility of menstrual health in the context of COVID vaccine testing would be a valuable addition to the manuscript. Menstruation is an essential aspect of women's health, yet it has frequently been disregarded or insufficiently considered in medical research. Highlighting this issue would contribute to raising awareness about the importance of inclusive research practices and promoting gender-sensitive approaches in healthcare.

I have carefully integrate these points into revised manuscript, emphasizing the impact of gender bias on medical diagnoses, the need for improved representation of women in research, and the significance of menstrual health visibility.

**Reviewer 1: Specific Comments**

**Author response to reviewer 1 comments regarding to the introduction:**

**•** ‘can disrupt the luteal phase, cause ovulation to stop, and lead to scanty infrequent periods, or oligomenorrhea’ – it sounds like they experience one or the other rather than oligomenorrhea is the medical term for what you’ve described. Rewrite for clarity.

• ‘because anxiety is associated with higher menstrual scores’ – what are menstrual scores?

• ‘menstruating people’ – use ‘menstruators’

• ‘in their study was funded that changes in menstrual volume were’ – do you mean found?

• ‘Finally, the investigates in their study’ – I’m not sure what investigates are

• ‘In another hand’ – should be ‘on the other hand’

**Author response:** Thank you for your thorough review of the manuscript. I have carefully revised the introduction to address the issue of unnecessary word repetition and potential misunderstandings. Furthermore, I have made the necessary corrections throughout the introduction based on your valuable comments.

**Author response to reviewer 1 comments regarding to the method:**

**Comment 1:** why was a population proportion of 50% chosen?

**Author response:** Thank you for your question regarding the choice of a population proportion of 50% in the study. I appreciate your inquiry, and I would be happy to provide an explanation.

In my research, the decision to use a population proportion of 50% was based on several considerations. **Firstly,** a proportion of 50% is often considered a conservative estimate in situations where there is limited prior knowledge or information about the variable being studied. By assuming a proportion of 50%, I aimed to ensure that the sample size would be large enough to capture any significant deviations from this estimate, thus increasing the statistical power of the study.

**Secondly,** choosing a population proportion of 50% allows for the most conservative estimation of sample size. This approach ensures that the study would have the ability to detect meaningful differences if they exist. By setting the proportion at 50%, essentially assuming that the variable under investigation has an equal likelihood of occurrence or presence in the population, thus maximizing the potential for identifying significant associations or effects.

**Lastly,** it is worth noting that the choice of a population proportion of 50% is common in various fields of research, particularly when investigating binary outcomes or proportions. This choice provides a balanced approach that does not introduce any preconceived bias into the analysis.

**While I acknowledge that specific studies or contexts may warrant different population proportions, I decision to use 50% in this particular study was primarily driven by the considerations outlined above.**

**I have added explain about the Population proportion 50% in the Estimation of sample size section in revised manuscript.**

**Comment 2:** What are the professional backgrounds of the ‘experts?

**Author response:** Thank you for your question regarding the professional backgrounds of the ‘experts. I have made revisions to the Method and Tool Data Collection section to provide more detailed information about the expert backgrounds involved in the study.

**Comment 3:** In the sample size you propose to interview 400 people and then later you say you interviewed 500 women?

**Author response:** Thank you for your question regarding the sample size. I have made revisions to the Method and Tool Data Collection section to provide more detailed information about sample size as the following:

The researcher conducted interviews with a total of 500 menstruators in order to achieve a matching sample, specifically consisting of 400 menstruators of reproductive age. It is important to note that during the participant selection process, 100 subjects were excluded from the study. Out of these, 90 subjects did not meet the inclusion criteria outlined for the study. Additionally, 10 subjects declined (refused) to participate in the study, citing personal reasons, lack of time, or a preference to not provide information about their health. These exclusions and refusals were carefully documented and taken into consideration during the data collection process. The sample size of 400 was determined after accounting for these exclusions and refusals, ensuring a representative and appropriate sample for the study.

**Author response to reviewer 1 comments regarding to the results:**

**Comment 1:** Women who participated in the study were in reproductive age’ you don’t need to repeat this you’ve already stated in methods.

**Author response:** Thank you for bringing this to my attention. I apologize for the oversight. In the revised version of the manuscript, I have removed the statement "Women who participated in the study were in reproductive age" from the result section.

**Comment 2:** ‘Regarding to socio-demographic data the (Table 1) shows that highest percentage (57.1%) (58.9%) of the study sample’ – Sorry I don’t understand this sentence ‘Above twenty (24.3%) of the participants had a primary schooling while’ – above 20 what? ‘Maternal status’ – you mean marital status.

**Author response:** Thank you for your comments regarding explain of table 1.

I sincerely apologize for the oversight in the initial version. In the revised version of the manuscript, I have thoroughly reviewed and corrected all the demographic data presented in Table 1. Furthermore, I have provided a clear and detailed explanation of the demographic information in the revised manuscript, **specifically highlighting the corrections made.**

**Comment 3:** Results – why have you chosen to highlight some stats but not others? The writing in the results should add something to the data that’s in the table, not just repeat it.

**Author response:** Thank you for you. **In the results section**, I aimed to provide a concise summary and interpretation of the key findings from our study. I acknowledge the reviewer's comment regarding the need for the written analysis to add value beyond the information presented in the table.

**To address this concern**, I have **revised the results section to provide more in-depth insights and explanations that complement the data presented in the table**. I have expanded upon the significance and implications of the highlighted statistics, explaining their relevance to the research objectives and providing additional context or interpretation where appropriate.

**Comment 4:** Could the results of the table 3 impacts on menstruation be instead presented in some form of visual graph so that the reader can really see the differences before and after covid/the vaccine. It’s harder to digest the information when it’s just numbers in a table.

**Author response:** Thank you for your suggestion regarding table 3. I would like to express my appreciation for your thoughtful review of my work. Your feedback and suggestions have been invaluable throughout this process. However, I wanted to kindly express my disagreement regarding the proposed change of converting Table 3 into a visual graph.

Table 3, as it stands, provides a concise and organized presentation of the data, allowing for easy reference and comparison.

**Comment 4:** ‘and about seventy (36.5%, 20.8%, 5.3%,3.5%, and 1.5% respectively)’ - can you make it clearer to the reader where these percentages come from – especially since they’re from the table after the text.

**Author response:** Thank you for you. I have revised the table 3 in the results section to provide more in-depth insights and explanations. **Specifically highlighting the corrections made.**

**Author response to reviewer 1 comments regarding to the discussion:**

**Comment 1:** Dysmenorrhea – give a definition for this

**Author response:** Thank you for your, In the revised version of the manuscript. I have defined and highlight it in the discussion section.

**Comment 2:** Did demographic data like age have any impact on the effects of the vaccine on their menstruation?

**Author response:** Thank you for you. I appreciate your suggestion to consider the impact of age on the effects of the vaccine on menstruation. However, it is important to note that my study did not specifically aim to assess the relationship between age and the vaccine's effects on menstruation.

While age is an important demographic factor, my study focused on other variables and did not include age as a specific variable of interest. I acknowledge that age could potentially be relevant to the effects of the vaccine on menstruation, and further research is warranted to explore this relationship in more depth.

**Comment 3:** Future studies are necessary’ – what would be the purpose of future studies?

**Author response:** Thank you for your valuable feedback. I agree that further research is necessary in this area. Future studies would serve several important purposes:

- Confirmatory evidence.
- Long-term effects: the present study have focused on short-term effects, but examining the long-term impact of the vaccine on menstruation is crucial. Future studies could investigate the duration and persistence of menstrual changes following vaccination.
- Subgroup analysis: Conducting subgroup analyses based on demographic factors, such as age, could help identify potential variations in the effects of the vaccine on menstruation.
- Comparative studies: Comparing the effects of different vaccine types or formulations on menstruation could be valuable in discerning whether observed effects are specific to a particular vaccine or a more general response to vaccination.

**Comment 4**: I’d like to see more explanations/discussion on why this was a shortcoming of those developing the vaccine i.e. what have been the detrimental impacts of these changes in menstruator’s periods, how this could be avoided in the future, and how scientists and healthcare professionals can warn people of these side effects.

**Author response:** Thank you for your insightful comments and suggestions. I appreciate the opportunity to further elaborate on these points in the revision manuscript.

I have provided a more in-depth discussion on the potential detrimental impacts of menstrual changes observed in individuals who have received the vaccine. This could include exploring the impact on menstrual regularity, duration, intensity of bleeding, or associated symptoms. I have review existing literature and relevant studies to provide a comprehensive overview of these effects.

**I have incorporated these important aspects into revised version of the manuscript, providing a comprehensive discussion on the shortcomings of vaccine development, the detrimental impacts of menstrual changes, strategies for avoiding such effects in the future, and the crucial role of scientists and healthcare professionals in communication.**

**Comment 5:** The section on cycle length in the discussion is very lengthy and doesn’t give much insight.

**Author response:** Thank you for you. I appreciate your perspective and understand your concern about the length of this section. I apologize if it didn't provide sufficient insight as desired. Based on your feedback, **I have made appropriate revisions to improve its clarity and relevance and highlight it in the revision manuscript.**

**Response to Reviewer 2**

This paper brings a global view on the association between the Covid vaccine and women's menstrual cycles. The focus on Iraqi women is an important contribution. However, there are some major issues with the paper:

Dear Reviewer,

Thank you for taking the time to review my manuscript. A Global Perspective with a Focus on Iraqi Women." I appreciate your valuable feedback and recognize the importance of your comments in improving the quality of my research. I have carefully considered your concerns and have outlined my responses below.

**Reviewer 2: General Comments**

**Comment 1:** The entire article has to be reviewed for grammar, typos, etc. The lack of attention to detail makes it very sloppy and make it seem as if the author does not care about the work. It's a sign of respect (for the work, the participants, the reviewers, and the reader) to make sure Covid is not spelled "Coved"

**Author response:** Thank you for your feedback and valuable suggestions regarding my manuscript. I have sent the manuscript to an English language expert for proofreading and correction. Additionally, I have rewritten the manuscript based on their feedback.

**Comment 2:** It's strange that there is only one author on this work, considering the magnitude of the endeavor and the involvement of several centers and experts. This reviewer would have expected a multi-disciplinary authorship team, with a gender representation of scientists and contributors.

**Author response:** Thank you for your feedback regarding the authorship of my work. I appreciate your perspective and understand your point regarding the magnitude of the eneavor and the involvement of several centers and experts. Allow me to explain the circumstances and address your concerns. While it is true that my study involved collaboration with multiple centers and experts, the decision to have a single author was made for specific reasons.

**Firstly**, as the primary researcher and investigator of this study, I have dedicated significant time, effort, and expertise to collect and analyze the data, ensuring the validity and reliability of the findings. Given the complexity of the topic and the need for consistent methodology throughout the study, having a single author provided a cohesive approach and minimized potential discrepancies.

**Secondly**, the decision for a single author does not diminish the collaborative nature of the study. It is important to highlight that the involvement of multiple centers and experts was instrumental in data collection, sample acquisition, and access to diverse populations. **The contributions of these collaborators have been duly acknowledged in the acknowledgments section of the paper.**

**Comment 3:** The paper could benefit from commentary on culture and menstruation in this part of the world; and why this work is important in the context of the Covid-19 pandemic and vaccines

**Author response:** Thank you for your insightful feedback on the paper. I appreciate your suggestion to include commentary on culture and menstruation in this specific region, as well as the importance of the present work in the context of the Covid-19 pandemic and vaccines. **I agree that addressing these aspects would enrich the paper and provide a more comprehensive understanding of the study's significance. I have incorporated these elements into the revised version of the manuscript.**

**Reviewer 2: Specifics Comments**

**Author response to reviewer 2 comments regarding to the introduction:**

**Comment 1-A:** This sentence "However, research hasn’t directly linked these changes to the COVID-19 vaccines. In fact, research into the way vaccines can impact menstruation is generally lacking" is immediately followed by this sentence, which puts the two at contradictory ends of the spectrum "The Medicines and Healthcare Products Regulatory Agency (MHRA) in UK

reported that between December 8, 2020 and September 8, 2021 there were 34,633

incidents of menstrual and vaginal bleeding reactions reported to them in relation to a

COVID-19 vaccine in Great Britain"

**Author response:** Thank you for drawing. I apologize for any confusion caused and appreciate the opportunity to address this issue.

The sentence stating that "research hasn't directly linked these changes to the COVID-19 vaccines" was intended to reflect the limited scientific literature available at the time of writing the manuscript. While there were anecdotal reports and individual accounts suggesting a potential association between COVID-19 vaccines and menstrual changes, the conclusive scientific evidence was still lacking.

However, I understand that the subsequent sentence referring to the report from the Medicines and Healthcare Products Regulatory Agency (MHRA) in the UK may have created confusion. The purpose of including this information was to highlight the existence of reported incidents of menstrual and vaginal bleeding reactions related to COVID-19 vaccines. The MHRA report serves as an important source of real-world data indicating potential associations, but it does not establish a direct causal link between the vaccines and the reported menstrual changes.

**I have revised the introduction section in general in the revised version of the manuscript.**

**Comment 1-B:** There is also AMPLE research on the Covid vaccines and female menstruation that we implore the author to review; including the Apple Women's Study. Infact, the author mentions this work in the Discussion. So why are they saying there is limited data on Covid vaccines and menstruation? This is not true, nor is it strong justification of the study.

**Author response:** Thank you for your understanding and acknowledgement of the availability of research on Covid vaccines and female menstruation, including the Apple Women's Study. I apologize for any confusion caused by the statement regarding limited data on Covid vaccines and menstruation in the manuscript.

**I have taken your feedback into account and made the necessary revisions to ensure that the paper accurately reflects the specific focus and scope of the study on the impact of Covid-19 vaccines on menstrual cycles in the Iraqi population. The revised manuscript will clearly state the objective of my study within the context of Iraqi women, addressing the specific gaps in knowledge in this region.**

**Comment 2:** With that said, a stronger case should be made for the focus on Iraqi women rather than the view that there is, globally, limited research on Covid and menstrual changes because that is not true and this entire paragraph needs to be reworked to focus on the study's population, the fact that the cross-sectional design does not get at the "short and long term" effects of the vaccine, the focus on Covid infection history/not, etc.

"The researcher thought society need this study because some women have

reported changes in their menstruation after receiving the COVID-19 vaccine,

including changes in duration, flow, and accompanying symptoms such as pain. In

another hand, the importance of the study as more people are vaccinated for COVID19, it is possible to gain a better understanding of the short- and long-term effects of

the vaccines. Scientific evidence could also help unvaccinated people understand

what, if any, menstruation-related side effects to expect from a COVID-19 vaccine.

Finally, the aim of the study to identify if have a connection between vaccination by

(COVID-19 vaccine) and changes in the menstrual cycle of women of reproductive"

**Author response:** Thank for you. I appreciate your feedback and emphasis on the need for a stronger case regarding the focus on Iraqi women in our study. In light of this, **I have reworked the paragraph to address the study's population, and the significance of investigating the relationship between COVID-19 vaccination and menstrual changes** **into the revised version of the manuscript.**

**Author response to reviewer 2 comments regarding to the method**

**Comment 1:** Is this true for Iraq or for ALL places in the world? If the latter, it's not a true statement. "The researcher selected the primary health care centres as the setting of the study because women of reproductive age visit primary health care centres for health services."

**Author response: Thank you for you.** The statement made in the study is specific to the context of the research and **does not apply universally to all places in the world**. The researcher selected primary health care centres as the setting for their study based on the policy of the Iraq Ministry of Health, where menstruator of reproductive age commonly visits these centers for health services in Kurdistan Region, Iraq.

**Comment 2:** What does this mean? "contaminated and uncontaminated COVID19 "

**Author response:** Thank you for you. Is mean infected and uninfected by COVID19. **I have changed it and highlight it in the method section in revision version manuscript.**

Comment 3: why this inclusion criteria? What is the biological premise? "received the COVID-19 vaccine no longer than 3 mounts included in the study"

**Author response:** Thank you for you. The biological premise behind the inclusion criterion of "received the COVID-19 vaccine no longer than 3 months" in a study investigating the effect of COVID-19 on the menstrual cycle is based on the hypothesis that the immune response triggered by the COVID-19 vaccine may potentially influence menstrual patterns.

The menstrual cycle is regulated by a complex interplay of hormones and immune system factors. Inflammation and immune responses can impact hormone levels and disrupt the normal menstrual cycle. COVID-19 itself has been associated with various systemic effects and inflammatory responses**. I have explained this point in the method section in the revised manuscript.**

**Comment 4:** But, for reliability estimated 400 women of reproductive age were included in the study" what reliability?

**Author response:** Thank you for you. Sample Size Reliability: the reliability or statistical power of the study based on the sample size. In research, a larger sample size generally provides more reliable and accurate results. Including 400 women of reproductive age in the study suggests that the sample size is sufficient to achieve a reasonable level of statistical power and reliability. A larger sample size reduces the likelihood of chance findings and increases the generalizability of the study's findings to the target population.

**I have added additional information regarding this point in the revised manuscript.**

**Comment 5:** Some details on why participants refused to participate in the study will be interesting...see comment on culture and menstruation.

**Author response:** Thank you for your valuable suggestions. Upon analysing these instances, two recurring themes emerged that shed light on the reasons behind their refusals. These themes include cultural considerations and concerns related to menstruation. **I have explained it in the method section in the revised manuscript.**

**Comment 6**: Grammar and sentence structure in sentences below. Also, aren't categorical variables also "numeric"? do you mean "continuous variables?" instead of numerical data?

"Categorical variables in numbers and percentages (%). Numerical data were analyzed

for normal distribution.

Author response: Thank you for you. I have corrected and rewrite the statistical analysis section in the revised manuscript.

**Author response to reviewer2 comments regarding to the results**

**Comment 1:** Recommend removing this phrase and getting to the point faster:

"Women who participated in the study were in reproductive age. All of them

received Covid-19 vaccine and their menstrual cycle were regular before received

Covid-19 vaccine. The study sample were free from hormonal disease and don't take

hormonal therapy."

**Author response:** Thank you for bringing this to my attention. I apologize for the oversight. In the revised version of the manuscript, I have removed the statement "Women who participated in the study were in reproductive age…….." from the result section.

**Comment 2:** Having trouble understanding this sentence "Regarding to socio-demographic data the (Table 1) shows that highest percentage (57.1%) (58.9%) of the study sample"and this too "Concerning COVID-19 infection, 63.8% of participants had contaminated. "

**Author response:** Thank you for your comments regarding explain of table 1. I sincerely apologize for the oversight in the initial version. In the revised version of the manuscript, I have thoroughly reviewed and corrected all.

Comment 3: This is great data; and would be good to see if any one of these vaccines was more associated with the changes reported versus others; especially since the discussion begins with a sentence about the vaccine types:"The majority of the study sample received Pfizer-BioNTech, Sinopharm, Johnson & Johnson's Janssen and AstraZeneca (76.5%, 10.8%, 8.5 and 4.0%, respectively), and all of them (100%) received two doses of Covid- 19 vaccine and the 79.5% of them was one month between the dosages "

**Author response:** Thank you for your feedback and suggestion to examine the potential associations between different vaccine types and the reported changes. We appreciate your interest in exploring this aspect of the data.

while the present study acknowledges the distribution of vaccine types among the participants, it is important to clarify that the primary focus is on examining the effects of COVID-19 vaccines on women in the reproductive age group. The mention of vaccine types in the discussion section was intended to provide context and describe the composition of the study sample accurately.

As stated in the introduction and objective of our study, our primary aim is to investigate the impact of COVID-19 vaccines on women in the reproductive age group, including potential effects on menstrual patterns. I did not design the study to compare the specific effects of different vaccine types on these outcomes. Therefore, exploring the associations between vaccine types and the reported changes falls beyond the scope of the research objectives.

**Comment 4:** SEVERAL typos on all the tables and headers and perhaps a number of these tables can be combined. Not very rigorous; merely descriptive and self-reported data.

**Author response:** Thank you for your valuable feedback on the manuscript. I have carefully considered your comments and made the necessary revisions to improve the quality of the tables and headers.

Regarding the issue of typos, I have thoroughly proofread the manuscript and have taken steps to ensure that all tables and headers are now free of errors.

**Comment 6:** Why was it important to collect data on vaginal redness/inflammation. The justification for this, as it relates to the vaccine is not provided and/or discussed anywhere ...Interesting variable to collect though!

**Author response:** Thank you for your valuable feedback regarding the importance of collecting data on vaginal redness/inflammation in relation to the vaccine. I appreciate your insights, and I have appropriately addressed this point in the discussion section of the revised manuscript.

**Author response to reviewer 2 comments regarding to the** **Discussion**

**Comment 1:** In the introduction author mentions "There is limited data on Covid vaccination and menstruation" but then have this paragraph in the discussion. So the premise of the research is invalidated...the introduction and justification need re-working as the current study is not adding anything new to the literature.

A systematic review study conducted by Nazir et al 2022, who found after receiving the second dose of the COVID-19 vaccine was a predictor of menstrual problems, after receiving the COVID-19 vaccine, women reported a significantly increased in the duration of their menstrual cycles compare to pre- COVID-19 vaccine status (12) in another hand, the finding of the current study is consistent with an Arabic Countries (Saudi Arabia, Egypt, Syria, Libya, and Sudan) study conducted by Amer et al. who have reported that the length of a menstrual duration varies by up to 8 days it is abnormal(10, 13).

**Author response:** Thank you for your comment regarding the introduction and justification of the research. I appreciate your feedback and would like to address your concerns.

Upon reviewing the feedback, I recognize that there may have been confusion in the way the introduction and discussion were presented. I apologize for any inconsistencies and assure you that the study aimed to contribute to the existing literature on the relationship between COVID-19 vaccination and menstruation.

**I have thoroughly reviewed and corrected all the inconsistencies in the introduction and discussion in the revised manuscript.**

**Comment 2:** The recall bias i) does not only affect those with menstrual irregularities but has to do with the study design and ii) the analyses should include a table with menstrual irregularities excluded.

**Author response:** Thank you for your feedback regarding recall bias and the suggestion to include a table with menstrual irregularities excluded in the analyses. **I appreciate your insights, and I have addressed these points in the revised manuscript.**

**Comment 3:** The discussion is not coherent and needs reworking. Again, a bit sloppy.

**Author response:** Thank you for your feedback regarding the coherence and overall quality of the discussion section. I apologize for any shortcomings in its organization and presentation. I acknowledge the importance of a clear and well-structured discussion to effectively convey the findings and implications of the study.

I have thoroughly rework the discussion section in the revised manuscript to ensure its coherence and logical flow.I have pay close attention to the organization of ideas, ensuring that each point is properly introduced, supported by evidence, and connected to the overall research objectives and significance.

Specifically, I have addressed the following areas to enhance the coherence of the discussion:

1- Clear objective: I have provided a concise and focused statement of the study's objective at the beginning of the discussion section to guide the reader and maintain a clear focus throughout.

2-Logical progression: I have carefully structured the discussion to present findings in a logical order, highlighting the most important results and their implications.

3- Integration of previous literature: I have strengthened the connection between our findings and the existing literature by more effectively integrating previous studies and their implications.

4-Limitations and future directions: I have provided a comprehensive discussion of the limitations of the study, including potential sources of bias, and areas for further investigation.

**Thank you for your time and consideration.**

**Sincerely,**

**Dr. Warda Hassan Abdullah**
