# Supplementary material for: Menstrual changes after COVID-19 vaccination among menstruators of reproductive age: A cross- sectional study from Erbil City, Iraq
Source: PLOS Glob Public Health. 2024 Apr 4;4(4):e0003079. doi: 10.1371/journal.pgph.0003079 (PMC10994343; doi:10.1371/journal.pgph.0003079)
Supplement: Attachment — Submitted filename: PLOS Review_II_19072023.docx [file pgph.0003079.s003.docx]

**19072023**

**Reviewer’s comments:**

This paper aims to establish an association between Covid vaccination and menstruation. Selecting an Iraqi city for data collection is a valuable consieration. Unfortunately, there are some major issues in the research conduct that restricted generating a meaningful and reliable evidence. Therefore, it is recommended to re-conduct the research and not to publish the findings. The issues are as follows:

- Literature review: It is unclear what data bases were searched for the literature review and how this search was unbiased or not selective. There is only one researcher involved and lack of contribution from a subject librarian may enhance the chance of information bias.
- Method:
  a. The study failed to describe how the experts were selected, how their opinion was collected in the development of the data collection tool.
  b. For the data collection, in-depth interview method was used but there is no indication of what language was used, whether it was recorded and transcribed, where it was stored, how and when it was anonymised. Also, there was no information around the nature of the data, was it all qualitative or there are some quantitative data as well? It was also absent in the description of data collection method if there were any quantifiable data around the degree of changes in menstruation.
  c. There was no mention of how the data had been managed in terms of storing and destroying. It was unclear where the hard and soft copies of data were stored, who were responsible for their maintenance and when they should be destroyed.
  d. There was no mention of GDPR.
  e. There was no qualitative data analysis and no explanation around what happened with the qualitative data collected by conducting interviews.
  f. Recruitment: a convenience sampling was used but there was inadequate description of how the participants were recruited, what were their selection criteria, who recruited them, whether there personal data were accessed and if so, then who accessed them, whether there was any permission from an appropriate authority around that etc.

Also it was unclear how 500 participants were interviewed and how 10 of them did not agree to share information.

Lack of these details of the methodology it is insufficient to allow the experiments to be reproduced

- Ethics:
  - It is not evident that the study is ethically sound. In the first meeting with the potential participants, the author/ researcher introduced the study to them. There was no Participants Information Sheet shared with the potential participants. As a result there was no scope for the participants to revisit the information about the study aim, expectation, time commitment, risks and benefit, impact etc. and decide on their participation.
  - Verbal consent was obtained from the participants and there is no information on whether it has been recorded, if so how and whether this was witnessed.
- Results: The manuscript has serious concerns around the methodology of data collection, data management and data analyses. As a result, the derived conclusions and results are not reliable. Moreover, absence of sub-group analysis made the claims much weaker.
