# Supplementary material for: Menstrual changes after COVID-19 vaccination among menstruators of reproductive age: A cross- sectional study from Erbil City, Iraq
Source: PLOS Glob Public Health. 2024 Apr 4;4(4):e0003079. doi: 10.1371/journal.pgph.0003079 (PMC10994343; doi:10.1371/journal.pgph.0003079)
Supplement: Attachment — Submitted filename: Response to Reviewers.docx [file pgph.0003079.s004.docx]

**Manuscript: PGPH-D-23-00059R1**

**Response to Reviewers**

**Dear Dr. Nazmul Huda**

Thank you very much for giving Me the opportunity to revise and resubmit the manuscript entitled ‘‘Menstrual Changes after COVID-19 Vaccination among Menstruators of Reproductive Age: A Cross- Sectional Study from Erbil City, Iraq’’. I deeply appreciate the time and effort that the academic editor and the reviewers devoted to provide comments and suggestions on my manuscript. I’m is grateful for their invaluable comments as they enriched and improved the manuscript.

I have carefully considered and responded to each comment made by the editor and the reviewers and I have successfully incorporated most of the suggestions into the revised manuscript.

The corresponding changes are highlighted “yellow” in the manuscript text in the revised file.

Thank you again for your consideration of my revised manuscript.

Sincerely,

Warda Hassan Abdullah

**Editor Comments**

Journal Requirements: In the ethics statement in the Methods, you have specified that verbal consent was obtained. Please provide additional details regarding how this consent was documented and witnessed, and state whether this was approved by the IRB.

**Author response:** Thank you for your question. I obtained approval from the ethical committee of the College of Nursing, Hawler Medical University, which granted permission to initiate the study. It is important to note that we do not have an Institutional Review Board (IRB), but we do have an ethical committee and Midwifery Scientific Committee at our College and University to oversee ethical considerations. The approval from this committees ensures that the study adheres to ethical guidelines and safeguards the rights and well-being of the participants.

In accordance with the ethics statement outlined in the Methods section of the study, verbal consent was obtained from all participants. The process of obtaining verbal consent" ensured both transparency and adherence to ethical guidelines.

To elaborate on the specifics of how verbal consent was documented, the following steps were taken:

1- A standardized verbal consent script was developed, as presented in the proposal of the study, and it was approved by the Scientific Midwifery Committee and the Ethical Committee at the College of Nursing, Hawler Medical University. This script provided a clear explanation of the study's purpose, procedures, risks, benefits, and the voluntary nature of participation."

2- Verbal Explanation: researcher administered the verbal consent process to participants. This involved verbally presenting the key points from the consent script (provided a clear explanation of the study's purpose, procedures, risks, benefits, and the voluntary nature of participation) ensuring that participants had a comprehensive understanding of the study.

3- Opportunity for Questions: After presenting the verbal consent information, participants were given the opportunity to ask any questions they had about the study. This ensured that any doubts or concerns were addressed before proceeding.

**Reviewers Comments**

**Response to Reviewer: 1**

**Reviewer #1:** Thank you for your revised version and taking our comments into consideration. You’ve clearly put in a lot of effort and the paper is looking great. So important to shed light on this topic in different contexts – I think it adds a lot of value to the discourse. I have just a few more changes that could be made if you agree. It was a pleasure to read and review.

Author response: Thank you for your kind words and for taking the time to review the revised version of the paper. I truly appreciate your positive feedback and your recognition of the effort I've put into addressing your comments.

**Reviewer 1: Comments:**

**Comment 1:** Some passive voice – consider changing to active voice e.g. the authors utilized a structured questionnaire rather than A structured questionnaire was utilized.

**Author response:** Thank you for your valuable feedback. I appreciate your suggestion to enhance the clarity and engagement of the writing by using active voice. I have revised the manuscript accordingly. For instance, in the mentioned sentence, I changed "A structured questionnaire was utilized" to "The author utilized a structured questionnaire." I have carefully reviewed the entire manuscript and made similar changes throughout to ensure a consistent use of active voice. This modification has not only improved the readability of the text but has also contributed to a more dynamic and engaging presentation of the research findings.

**Comment 2:** Maybe just specify that the menstruators are of reproductive age just once, and then continue to refer to them just as menstruators as ‘menstruators of reproductive age’ is a bit clunky to keep repeating.

**Author response:** Thank you. In response to your suggestion, I will introduce the term "menstruators of reproductive age" at the beginning of the relevant section to establish the context and ensure that the reader understands the specific demographic being referred to. After this initial clarification, I will then use the term "menstruators" in subsequent references to avoid the clunky repetition of "menstruators of reproductive age."

**I have made the necessary change throughout the in manuscript. based on your valuable comment.**

Comment 3:

**Author response:** Thank you for bringing up this crucial point and underscoring the significance of inclusivity in the manuscript. Your insight is greatly appreciated and aligns with our commitment to respectful and comprehensive terminology.

In light of your suggestion, I will include a brief explanation about the term "menstruator" in the **methods section** of the manuscript. I will clarify that the term is chosen to be inclusive of all individuals who experience menstruation, regardless of gender identity. This explanation will ensure that readers who may not be familiar with the term understand its purpose and the broader context of inclusivity it represents.

**Comment 4: Would another key word be COVID-19** **vaccine?**

**Author response:** Thank you for your insightful comment. I agree that the inclusion of "COVID-19 vaccine" as a keyword is highly relevant to the study. Given the current global context and the significance of vaccination efforts, this keyword aligns well with the research focus. I will certainly incorporate "COVID-19 vaccine" as one of the keywords associated with the manuscript.

**Comment 5:** Title – should be menstruators (plural)

**Author response:** Thank you for your comment regarding the title. The researcher changes the menstruator to menstruators in the title of study.

**Comment 6:** I still feel that there are a few areas of repetition – for example in your introduction you repeat the aim of the study twice, and then again in the first line of your method, and then in the discussion – maybe limit this to just twice throughout the paper.

**Author response:** Thank you. I sincerely appreciate your meticulous review of the manuscript and your feedback regarding areas of repetition.

Upon reviewing your observation, I recognize the instances of repeated statements regarding the aim of the study in the **introduction, method, and discussion** sections and corrected it.

**Comment 7:** You explain about using a slightly larger sample size than 400 – can you please state that it was 500 when explaining this.

Author response: Thank you, I appreciate the reviewer's careful attention to the manuscript and the feedback regarding the sample size explanation. I apologize for any confusion and would like to clarify the discrepancy.

In the study, I initially aimed to achieve a sample size of 400 menstruators. To ensure the robustness of the findings and account for potential attrition during the research process, I decided to conduct interviews with a larger cohort of 500 menstruators. This approach was undertaken as a precautionary measure to safeguard against any potential loss of statistical power due to participant exclusions or withdrawals.

As explained in the manuscript, a total of 100 subjects were excluded from the study during the participant selection process. Among these exclusions, 90 subjects did not meet the predefined inclusion criteria, and an additional 10 subjects declined to participate for various reasons. The final sample size used for our analysis was indeed 400 menstruators, as initially intended**.**

**Comment 8:** I would explain that dysmenorrhea is specifically painful menstrual cramps, rather than just menstrual pain. Also, you already explain its meaning once, no need to repeat.

**Author response:** Thank you for you. In response to your earlier suggestion to define dysmenorrhea, I included an explanation to ensure that readers, regardless of their familiarity with the term, can understand its specific meaning within the context of our study. We understand and respect your point that the term "dysmenorrhea" refers to painful menstrual cramps rather than just general menstrual discomfort. We aimed to emphasize this distinction to accurately portray the nature of the condition under investigation.

I apologize for any redundancy that may have arisen due to repetition of the definition. **I will review the manuscript again to ensure that the definition of dysmenorrhea is presented in a clear and concise manner, without unnecessary repetition.**

**Comment 9:** I know it’s extra work but perhaps some of the results could be visually represented by a graph – it would make it easier for the reader to see the differences before and after the vaccine. The tables are great but it’s a lot of numbers to digest

**Author response:** Thank you for you. I genuinely appreciate your thoughtful suggestion regarding the inclusion of graphs to visually represent the results of the study. Your input underscores the importance of providing a clear and accessible presentation of the findings. I value your commitment to enhancing the readability of the manuscript.

While I acknowledge the benefits that graphs can bring in terms of simplifying the visualization of differences before and after the vaccine, I have chosen to present the results using tables for specific reasons. My decision was primarily driven by the complexity and multifaceted nature of the data I am dealing with. Given the nuanced relationships I am exploring, tables provide a comprehensive and detailed depiction of the results, ensuring that every aspect of the findings is captured accurately.

I recognize that graphs can be more intuitive for certain types of data presentation, and I truly appreciate your suggestion in this regard. However, I believe that the comprehensive and precise representation provided by the tables aligns well with the intricacies of the study.

**Comment 10:** I find the discussion a little bland – it is mainly presenting the results again but comparing them with other studies. I love this point in your conclusion – ‘’The menstrual cycle is an important indicator of menstruator health, and disruptions in its regularity can signal underlying conditions. Therefore, it is essential to increase awareness among healthcare professionals and menstruator about potential menstrual abnormalities following Covid19 vaccination.’’ Perhaps you could introduce and expand on this a little bit more in the discussion – and get more into why are these findings important. From my view they’re important because (a) it shows the lack of consideration of menstruators and menstruation in public health, (b) it may have caused unnecessary physical discomfort and stress and (c) your concluding point that we should be aware of these side-effects so people can prepare for them and understand what’s happening in their bodies.

**Author response:** Thank you for your feedback and suggestions! I agree that discussing the importance of the findings and their implications for public health, menstruators' well-being, and awareness is crucial. I will revise the discussion section according to your comments in the **revision manuscript.**

**Response to** **reviewer 3 comments**

**Reviewer 3#**

This paper aims to establish an association between Covid vaccination and menstruation. Selecting an Iraqi city for data collection is a valuable consieration. Unfortunately, there are some major issues in the research conduct that restricted generating a meaningful and reliable evidence. Therefore, it is recommended to re-conduct the research and not to publish the findings. The issues are as follows:

**Response to Comment: Research Conduct and Findings Reliability**

Thank you for you. I sincerely appreciate the time and consideration you have invested in reviewing the paper that explores the potential association between Covid vaccination and menstruation. I acknowledge your valuable feedback and the concerns you have raised about the research conduct and the reliability of the findings.

I am committed to producing rigorous and trustworthy research, and your feedback provides me with an opportunity to critically assess and enhance the study. I will address the major issues you have highlighted:

**1. Data Collection Location: Iraqi City Selection**

I thank you for recognizing the value in selecting an Iraqi city for data collection. The decision to focus on a specific location was intended to provide insights into a localized context and potentially identify region-specific trends. However, I understand that this approach might have limitations in terms of generalizability.

**2. Research Conduct and Reliability Issues:**

I greatly appreciate your concerns about the research conduct and the reliability of the findings. In light of this, I am conducting a thorough review of the research methodology and data analysis to address the issues you have pointed out. I recognize that generating meaningful and reliable evidence is paramount, and I am taking your recommendations seriously.

**Next Steps:**

**To ensure the credibility of the research, I am taking the following steps:**

- Methodology Review: I am reassessing the research methodology to identify and rectify any potential biases or limitations that might have impacted the reliability of the findings.
- Data Verification: I am re-evaluating the data collection procedures and exploring options for independent verification of the collected data to enhance the accuracy and validity of the results.
- Statistical Analysis: I am collaborating with experts in statistical analysis to ensure the integrity of the data interpretation and conclusions**.**

**Comment 1 Literature review:**

It is unclear what data bases were searched for the literature review and how this search was unbiased or not selective. There is only one researcher involved and lack of contribution from a subject librarian may enhance the chance of information bias.

**Author response:**

**Response to Comment: Unclear Database Search**

Thank you for your, I appreciate the reviewer's valuable feedback regarding the literature review. I apologize for any confusion. In the manuscript I databases, Specifically, I used well-established academic databases such as PubMed, Scopus, and Google Scholar.

**Response to Comment: Sole Researcher Involvement**

Thank you for your, I appreciate the reviewer's inquiry regarding the presence of a single researcher in this study. The inclusion of only one researcher in this study aligns with a specific requirement within our institution's academic progression process, which mandates the completion of independent studies. In compliance with this institutional guideline, the study was undertaken by a sole researcher.

The decision to have a single researcher is in adherence to the academic objectives of our institution, emphasizing the development of individual research skills, critical thinking, and the ability to autonomously execute research projects. It provides researcher with an opportunity to delve deeply into their chosen subject matter and enhance their academic growth through self-directed exploration.

It's important to note that while only one researcher was directly involved in the study, the research process itself was not isolated. Continuous interactions with experts helped shape the study's design, methodology, and interpretation of results.

**Comment 2: Method:**

**a.** The study failed to describe how the experts were selected, how their opinion was collected in the development of the data collection tool.

**Author response:** Thank you for you. **Response to Comment: Expert Selection and Data Collection Tool Development**

Thank you for highlighting the need for clarity in the expert selection process and data collection tool development. **I am valuing your input and will address these aspects in the revised manuscript:**

**a. Expert Selection:**

In response to your concern, I will provide a detailed explanation of the expert selection criteria in the revised manuscript. Experts were chosen based on their academic accomplishments and professional experience, ensuring diverse perspectives.

**b. Data Collection Tool Development:**

To enhance transparency, I will describe the methodology I am employed for gathering expert opinions in tool development. This process involved preliminary consultations, questionnaire drafting, expert feedback integration.

**Comment 3: Method**

**b-** For the data collection, in-depth interview method was used but there is no indication of what language was used, whether it was recorded and transcribed, where it was stored, how and when it was anonymised. Also, there was no information around the nature of the data, was it all qualitative or there are some quantitative data as well? It was also absent in the description of data collection method if there were any quantifiable data around the degree of changes in menstruation.

**Author response:** Thank you for your feedback on the data collection methods. To clarify:

**Data Collection Method:** I apologize for the oversight. In the quantitative study, I utilized a paper-based questionnaire and during the interview’s participants' responses were recorded directly on the questionnaire sheets.

**Anonymization and Storage:** All questionnaire responses were anonymized by omitting personal identifiers. The collected data was securely stored in protected electronic files.

**Nature of Data:** the study primarily involved quantitative data collection through structured questionnaire responses. I focused on gathering participants' insights on the association between Covid vaccination and menstruation.

**I appreciate your insights, and these clarifications will be included in the revised manuscript. Your feedback contributes to the transparency and accuracy of the research**

**Comment 4**: Method

c- There was no mention of how the data had been managed in terms of storing and destroying. It was unclear where the hard and soft copies of data were stored, who were responsible for their maintenance and when they should be destroyed.

**Author response:** Thank you for your feedback on the Data Management, Storage, and Destruction. I will add information about this section in the 'Ethical Considerations' subsection of the Methodology into the revised version of the manuscript.

**Comment 5: method**

d- There was no mention of GDPR.

**Author response:** Thank you for your question. I'm sorry and apologize for not being entirely clear about what you mean by GDPR.

If you're referring to the General Data Protection Regulation, I did mention data considerations in the 'Ethical Considerations' section."

**Comment 6 method:**

e- There was no qualitative data analysis and no explanation around what happened with the qualitative data collected by conducting interviews.

**Author response:** Thank you for your comment regarding the absence of qualitative data analysis and the explanation needed for the interviews conducted in the study.

the study is fundamentally quantitative in nature. The term "interviews" referred to a structured process to assess participants' alignment with study criteria and their accurate completion of the questionnaire sheet. These conversations were not for qualitative data collection or analysis, but to ensure participants' suitability for the quantitative research.

**I apologize for any confusion and appreciate your diligence in seeking clarification. the focus was solely on quantitative data collection and analysis to address the research questions.**

**Comment 7 method:**

f- Recruitment: a convenience sampling was used but there was inadequate description of how the participants were recruited, what were their selection criteria, who recruited them, whether there personal data were accessed and if so, then who accessed them, whether there was any permission from an appropriate authority around that etc.

Also it was unclear how 500 participants were interviewed and how 10 of them did not agree to share information.

**Author response:** Thank you for your valuable comments on recruitment and participant information sharing. Here's how we address these concerns:

Recruitment Process:

I employed convenience sampling, selecting accessible participants. In the revised manuscript, I will clarify the selection criteria used.

Participant Data Handling:

Participants' data were handled with strict adherence to ethics and data privacy regulations. Only authorized researchers accessed data with proper permissions.

Participant Declination:

At the beginning of the study, 10 subjects declined to participate, citing personal reasons, lack of time, or a preference not to provide health information. **I will provide a clear explanation in the revised manuscript.**

**Comment 8 method**: Lack of these details of the methodology it is insufficient to allow the experiments to be reproduced

**Author response:** Thank you for highlighting the importance of reproducibility in the study's methodology. I recognize the need for greater clarity to facilitate replication. I will enhance the methodology section and clarify and explain your comments one by one.

**Comment 9 Ethics:**

**- It is not evident that the study is ethically sound. In the first meeting with the potential participants, the author/ researcher introduced the study to them. There was no Participants Information Sheet shared with the potential participants. As a result there was no scope for the participants to revisit the information about the study aim, expectation, time commitment, risks and benefit, impact etc. and decide on their participation.**

**Verbal consent was obtained from the participants and there is no information on whether it has been recorded, if so how and whether this was witnessed.**

**Author response:** Thank you for emphasizing the significance of reproducibility in the study's methodology. I acknowledge the requirement for enhanced clarity to accurately convey the processes and procedures involved. I will explain it more **in the revised Manuscript**

**The researcher has taken significant steps to address the ethical concerns raised by providing detailed and comprehensive information to potential participants. A thorough Participants Information Sheet has been prepared, outlining essential aspects such as the study's objectives, research approach, participant expectations, potential risks, benefits, and other pertinent details. This information has been thoughtfully translated into the local language (Kurdish) to ensure clarity and accessibility.**

**During the initial engagement with potential participants, the researcher has prioritized transparent communication. Emphasis has been placed on the pivotal role of informed consent, and the contents of the Participants Information Sheet have been meticulously explained. This approach guarantees that participants are fully cognizant of the implications of their involvement and enables them to ask questions and seek clarifications as needed. This active dialogue fosters an environment where potential participants can express any concerns they may have about the study's procedures.**

Moreover, the researcher has underscored the voluntary nature of participation, highlighting those individuals possess the autonomy to withdraw from the study at any point without incurring any adverse consequences. This assurance reinforces the ethical principle of respect for participants' rights and choices.

**Comment 10 Results:** The manuscript has serious concerns around the methodology of data collection, data management and data analyses. As a result, the derived conclusions and results are not reliable. Moreover, absence of sub-group analysis made the claims much weaker.

**Author response:** Thank you for your thorough review of our manuscript titled "Menstrual Changes after COVID-19 Vaccination among Menstruators of Reproductive Age: A Cross- Sectional Study from Erbil City, Iraq " I sincerely appreciate your time and valuable feedback, which has provided me with an opportunity to address the concerns you raised. I have carefully considered your comments and have made comprehensive revisions to enhance the quality and reliability of the study.

**Thank you for your time and consideration.**

**Sincerely,**

**Warda Hassan Abdullah**
