# Supplementary material for: Menstrual changes after COVID-19 vaccination among menstruators of reproductive age: A cross- sectional study from Erbil City, Iraq
Source: PLOS Glob Public Health. 2024 Apr 4;4(4):e0003079. doi: 10.1371/journal.pgph.0003079 (PMC10994343; doi:10.1371/journal.pgph.0003079)
Supplement: Attachment — Submitted filename: review_plosone.docx [file pgph.0003079.s005.docx]

In this study, the authors investigated the effect of covid 19 vaccination on menstrual cycle. The research question is very popular nowadays because of many unknowns abot covid 19 vaccination and the authors ver well explained the need of this research especillay in Iraq. I have a few suggestions and questions;

1. The authors used the term “menstruator” for women menstrating regulary but “Menstruators is an inclusive term for all people that menstruate, which includes cisgender women, transgender men, non-binary and intersex people” and this term is natural for gender. I suggest the authors to add a sentence to material method or result section if there is transgender men or intersex people among the participants.
2. Is there a difference in terms of menstrual irregularities between theinfected and uninfected groups?
3. Is there a need of table 5? It would be easy to follow the tables if the authors could add a p value for the difference between groups for every sign and symptoms in table 4 and table 3
4. It is unclear whether the interviewed women took anticoagulants during and after their infection, as this could affect the duration of menstruation and the amount of blood lost. This question should be included in the questionnaire
