# Supplementary material for: Menstrual changes after COVID-19 vaccination among menstruators of reproductive age: A cross- sectional study from Erbil City, Iraq
Source: PLOS Glob Public Health. 2024 Apr 4;4(4):e0003079. doi: 10.1371/journal.pgph.0003079 (PMC10994343; doi:10.1371/journal.pgph.0003079)
Supplement: Attachment — Submitted filename: Response to Reviewers.docx [file pgph.0003079.s006.docx]

**Manuscript: PGPH-D-23-00059R2**

**Response to Reviewers**

**Dear Dr, Avanti Dey**

Thank you very much for giving Me the opportunity to revise and resubmit the manuscript entitled ‘‘Menstrual Changes after COVID-19 Vaccination among Menstruators of Reproductive Age: A Cross- Sectional Study from Erbil City, Iraq’’. I deeply appreciate the time and effort that the academic editor and the reviewers devoted to provide comments and suggestions on my manuscript. I’m is grateful for their invaluable comments as they enriched and improved the manuscript.

I have carefully considered and responded to each comment made by the reviewers and I have successfully incorporated most of the suggestions into the revised manuscript.

The corresponding changes are highlighted “yellow” in the manuscript text in the revised file.

Thank you again for your consideration of my revised manuscript.

Sincerely,

Warda Hassan Abdullah

**Reviewers Comments**

**Response to Reviewer: 1**

**Reviewer #1: The researcher has largely addressed my comments. I just have a few more on methods, ethics and the discussion.**

**Author's response:** I appreciate your positive feedback and the time you dedicated to reviewing the updated version of the paper. I have invested my efforts in addressing your comments.

**Reviewer 1: Comments:**

**Comment 1:** Method and tool data collection: Line 201 – where was the data securely stored? You go on to explain this in data management but you can indicate it here/make note that you will go on to explain in the following section. How was it anonymized?

**Author response:** Thank you for your feedback. I will make the necessary revisions to the Method and Tool Data Collection section (line 201). Highlighted “yellow” in the manuscript text in the revised file.

**The collected data underwent secure storage through the encoding of questionnaire sheets to uphold confidentiality. Comprehensive measures were taken to ensure the secure storage and management of both hard and electronic copies. Physical questionnaire sheets, constituting hard copies, were placed in a designated, locked cabinet with restricted access. Simultaneously, electronic copies were safeguarded on a password-protected server, exclusively accessible to authorized researcher. In alignment with ethical guidelines and the protection of participants' confidentiality, a well-defined data destruction process was instituted. Following the publication of the study, all hard copies will be securely shredded to prevent unauthorized access. Similarly, electronic copies stored on the password-protected server will be deleted.**

**Comment 2:** Ethical considerations: Line 230 – who witnessed the verbal consent being given aside from the researcher?

**Author response:** Thank you for your inquiry. The verbal consent, aside from the researcher, was observed by the healthcare provider and participating relatives present during the data collection at the primary healthcare centers where the research was conducted.

**Comment 3: Line 243 – when will the copies be shredded? What is meant by study’s completion – once the paper is published?**

**Author response:** Thank you for your question. The copies of the data will be securely shredded once the study is published. By 'study's completion,' I am indeed referring to the publication of the paper. I have changed **'study’s completion'** to **'study's published'** in the revised manuscript and highlighted it.

**Comment 4:** **Discussion: The researcher did not address the comment of adding into the discussion that the results demonstrate the lack of consideration of menstruators and menstruation in public health, namely not using enough menstruators in drug trial studies (reference book ‘Invisible Women by Caroline Criado-Perez) or considering menstruation as a public health concern.**

**Author response:** Thank you for your comments. In response, **I have revised the discussion section**, incorporating your suggestions to emphasize the focus on menstruators and menstruation in public health. Additionally, I have referenced the book 'Invisible Women' by Caroline Criado-Perez in the revised manuscript

**Response to** **reviewer 4 comments**

**Reviewer #4**

**Reviewer #4: In this study, the authors investigated the effect of covid 19 vaccination on menstrual cycle. The research question is very popular nowadays because of many unknowns about Covid 19 vaccination and the authors ver well explained the need of this research especially in Iraq. I have a few suggestions and questions;**

**Author response**: Thank you for your positive feedback and for recognizing the relevance of the research question in the context of COVID-19 vaccination and menstrual cycles. I appreciate your interest in the study. I will follow your suggestions, address all comments, and attempt to incorporate your recommendations where possible. **I will highlight these changes in the revised manuscript.**

**Comment 1: The authors used the term “menstruator” for women menstrating regulary but “Menstruators is an inclusive term for all people that menstruate, which includes cisgender women, transgender men, non-binary and intersex people” and this term is natural for gender. I suggest the authors to add a sentence to material method or result section if there is transgender men or intersex people among the participants.**

**Author response:** Thank you for your insightful comment. In response to Reviewer #1's initial suggestion, I replaced the term 'Women of Reproductive Age' with 'Menstruators of Reproductive Age' throughout the manuscript.

The following is the Reviewer comment:

Reviewer #1: Comment 1: With recognition of those with gender-nonconforming identities such as transgender men and some non-binary persons using the term women to indicate those that menstruate is not inclusive. Please instead use the term ‘menstruator’ of reproductive age. This could even be turned into an acronym MORA. In the new definition for Menstrual Health this term is used along with a justification of using gender inclusive terms if you’d like a reference https://www.tandfonline.com/doi/full/10.1080/26410397.2021.1911618.

**As per the suggestion, a sentence has been added to the results section of the revised manuscript to explicitly state that there were no transgender men or intersex people among the study participants.**

**Comment 2:** **Is there a difference in terms of menstrual irregularities between the infected and uninfected groups?**

**Author response:** Thank you for your question. **The primary objective of my study is to investigate the relationship between COVID-19 vaccination and menstrual changes among menstruators in Iraqi society**. It is not within the scope of my study to identify differences in terms of menstrual irregularities between the infected and uninfected groups. Thank you for your question again.

**Comment 3:** **Is there a need of table 5? It would be easy to follow the tables if the authors could add a p value for the difference between groups for every sign and symptoms in table 4 and table 3**.

**Author response: Thank you for your valuable suggestion. I appreciate your feedback on the tables. To enhance clarity and facilitate a better understanding of the results, I have revised Tables 3 and 4 to include p values for the differences between groups for each sign and symptom. Additionally, after careful consideration, I have decided to omit Table 5 as I believe the inclusion of p values in Tables 3 and 4 adequately addresses the need for easy interpretation of the data. I hope these modifications contribute to the overall readability and comprehensibility of the present study findings. Your input has been instrumental in improving the presentation of the results, and I thank you for your thoughtful recommendation.**

**Comment 4: It is unclear whether the interviewed women took** **anticoagulants during and after their infection, as this could affect the duration of menstruation and the amount of blood lost. This question should be included in the questionnaire**

**Author response:**

Thank you for your comment. Regarding the inclusion and exclusion criteria of our sample, all participants reported being free from anticoagulants. This information has been clarified in the revised manuscript. I appreciate your attention to detail and your helpful suggestions.

Participants with major psychiatric disorders, hormonal diseases, or those receiving hormonal therapy**, as well as anticoagulant therapy during and after their infection**, were excluded from the study to ensure the clarity and specificity of the results

**Thank you for your time and consideration.**

**Sincerely,**

**Warda Hassan Abdullah**
