# Supplementary material for: Menstrual changes after COVID-19 vaccination among menstruators of reproductive age: A cross- sectional study from Erbil City, Iraq
Source: PLOS Glob Public Health. 2024 Apr 4;4(4):e0003079. doi: 10.1371/journal.pgph.0003079 (PMC10994343; doi:10.1371/journal.pgph.0003079)
Supplement: Attachment — Submitted filename: Response to Reviewers.docx [file pgph.0003079.s007.docx]

**Manuscript: PGPH-D-23-00059R3**

**Response to Reviewers**

**Dear Dr. Avanti Dey,**

Thank you very much for giving Me the opportunity to revise and resubmit the manuscript entitled ‘‘Menstrual Changes after COVID-19 Vaccination among Menstruators of Reproductive Age: A Cross- Sectional Study from Erbil City, Iraq’’. I deeply appreciate the time and effort that the academic editor and the reviewers devoted to provide comments and suggestions on my manuscript. I’m is grateful for their invaluable comments as they enriched and improved the manuscript.

I have carefully considered and responded to each comment made by the reviewers and I have successfully incorporated most of the suggestions into the revised manuscript.

The corresponding changes are highlighted “yellow” in the manuscript text in the revised file.

Thank you again for your consideration of my revised manuscript.

Sincerely,

Warda Hassan Abdullah

**Editor Comments**

**Comment 1: In the ethics statement in the Methods, you have specified that verbal consent was obtained. Please provide additional details regarding how this consent was documented and witnessed, and state whether this was approved by the IRB.**

**Author response:** I have made several additions to the manuscript. First, I have included detailed information regarding participant consent in the ethics statement. Furthermore, I obtained approval from the ethical committee of the College of Nursing, Hawler Medical University, which granted permission to initiate the study. It is important to note that we do not have an Institutional Review Board (IRB), but we do have an ethical committee at our College and University to oversee ethical considerations. The approval from this committee ensures that the study adheres to ethical guidelines and safeguards the rights and well-being of the participants.

In the present study, verbal informed consent was obtained from all the menstruators who participated. This consent process took place during the initial gathering at the primary health care centers, where the researcher explained the study's purpose and details to the participants and invited them to provide informed consent verbally. Verbal consent was chosen as the preferred method due to its greater acceptance in our society, as individuals generally exhibit hesitancy towards giving written consent.

**Comment 2:** P**lease review your reference list to ensure that it is complete and correct. If you have cited papers that have been retracted, please include the rationale for doing so in the manuscript text, or remove these references and replace them with relevant current references. Any changes to the reference list should be mentioned in the rebuttal letter that accompanies your revised manuscript. If you need to cite a retracted article, indicate the article’s retracted status in the References list and also include a citation and full reference for the retraction notice.**

**Author response:** Thank you for your message regarding the review of the reference list. I appreciate your diligence in ensuring the completeness and accuracy of my manuscript.

Upon reviewing the reference list, I have taken the comprehensive review: I have carefully examined each reference to ensure its relevance, accuracy, and current status.

**Reviewers Comments**

**Response to Reviewer: 1**

**Reviewer #1: Thanks for this next draft following our comments – I can see that you’ve taken much of what I’ve said into consideration and the paper is looking much better. I still have a few minor comments to address but apart from that I’m happy with how it is. Well done.**

**Author's response:** Thank you very much for your encouraging feedback on the revised draft of my manuscript. I sincerely appreciate your time and effort in providing valuable comments and suggestions.

I delighted to hear that you have noticed the changes made based on your previous feedback and that the paper is looking much better to you. Your guidance has been instrumental in enhancing the quality of my work, and I grateful for your thorough review.

I will carefully address the remaining minor comments you have and ensure that they are appropriately incorporated into the manuscript. Your continued support and constructive feedback are invaluable to me.

**Reviewer 1#: Comments**

**Comment 1:** **Introduction**

**o I still feel the paper is a bit repetitive in parts. Good to be clear and guide the reader but I think you state the objective too many times. For starters you could probably delete:**

** Introduction - ‘The primary objective of this study is to investigate the relationship between COVID19 vaccination and menstrual changes among menstruators in Iraqi society’**

** Discussion - ‘Menstrual Changes after COVID-19 Vaccination among Menstruators of Reproductive Age’**

**Author response:** Thank you for your feedback on the repetitiveness observed in certain sections of my manuscript. I appreciate your insightful observation and your suggestion to streamline the content for improved clarity and readability.

Introduction: I have removed the explicit statement related to the objective in the introduction part, as you suggested.

**Highlighted “yellow” in the manuscript text in the revised file.**

Discussion: Similarly, I will omit the redundant statement, "Menstrual Changes after COVID-19 Vaccination among Menstruators of Reproductive Age," from the Discussion section.

The below part has been removed from the Discussion section

The present study, examining Menstrual Changes after COVID-19 Vaccination among Menstruators of Reproductive Age, provides valuable insights into the potential effects of vaccination on menstrual health. The findings reveal a significant prevalence of alterations in menstrual patterns post-vaccination, highlighting a previously underexplored aspect of the COVID-19 vaccination experience in Iraq.

By eliminating these repetitive statements, I aim to enhance the flow and coherence of the manuscript while maintaining clarity and guiding the reader effectively.

**Comment 2: General**

**o Still areas where passive voice is used – would be great if the whole paper was in active voice**

**o Sometimes you’re missing full stops**

**o A few grammatical mistakes dotted throughout – please revise**

**Thank you for taking the time to review our manuscript and providing valuable feedback. I have carefully considered your comments and made the necessary revisions to address the issues raised.**

**Author response:** Regarding the use of passive voice, I have made concerted efforts to transition the entire paper to active voice to enhance clarity and engagement. I have carefully reviewed each section to ensure that active voice is consistently used throughout the manuscript.

Additionally, I have thoroughly proofread the manuscript to address missing full stops and correct any grammatical mistakes. I highlighted all changes in the revised manuscript.

**Comment 3: Methods**

**o I’m still a bit confused about the sample size calculation – had you planned for 500 or 400 participants? It seems convenient that you planned 500 with the knowledge that 100 would drop out to leave you with 400. You discuss sample size multiple times throughout the methods – would be neater to just have one section on it.**

**Author response:** Thank you for your insightful comment regarding the sample size calculation in the Methods section of my manuscript.

Initially, I planned for a final sample size of 400 participants, as indicated in the estimated sample size. However, to ensure that I reached this target number of participants who fit the inclusion and exclusion criteria, I anticipated potential dropouts and accounted for them in planning process. This is why I aimed to interview 500 individuals initially.

**Comment 4:** Results

o ‘more than half’ – not scientific be specific – could say majority or just actual percentage

o Duration of period - you mean full menstrual cycle length. The word period is also used to denote the days of bleeding so this could be misleading

o P value of exactly 0? Wow.

o You don’t need to explain what dysmenorrhea is more than once

**Author response:** Thank you for your feedback on the Results section of my manuscript

1- I appreciate your suggestion to avoid using qualitative terms like "more than half" and to instead provide specific percentages or use terms like "majority." I have revised the text accordingly to your suggested.
2- appreciate your observation regarding the potential ambiguity in the term "duration of period." You are correct that "period" can refer to both the days of bleeding (menstruation) and the entire menstrual cycle length. To clarify,

*when I refer to "duration of period" in the present study, I mean the days of bleeding.

*when I refer to ''duration between the periods" in the present study. I mean the length of the full menstrual cycle.

I highlighted all changes in the revised manuscript.

3- I understand your surprise at seeing a p-value of exactly 0. Allow me to clarify: in statistical analysis, a reported p-value of 0 signifies that the observed result is extremely improbable to have occurred by chance alone. It indicates a highly significant result, supporting the conclusions drawn from our analysis. I hope this clarifies any confusion.
4- I acknowledge the discrepancy in feedback from previous reviewers. While one reviewer suggested adding an explanation of dysmenorrhea, you have correctly pointed out that it may not be necessary to include this explanation more than once.

To address this, I will ensure that the explanation of dysmenorrhea is included only where it is essential for understanding and clarity. I have avoided unnecessary repetition to maintain conciseness and readability in the manuscript.

**Comment 5: Table 4**

**1- Would it be good to give us the % change? Perhaps the P value is enough.**

**2- why haven’t you put in the P values for the last 3 symptoms?**

**Author response:** Thank you for your comments.

1- I opted not to include the % change for several reasons. Firstly, providing only the % change without additional context may not offer a comprehensive understanding of the data. Additionally, including both % change and P values could lead to redundancy and clutter in the presentation of the results.
2- As for the absence of P values for the last 3 symptoms, I apologize for the oversight. I have ensured that the P values for these symptoms are included in the subsequent analysis to provide a more thorough interpretation of the results.

**I have added the P-values of the last 3 symptoms in the revised manuscript**

**Comment 6: Discussion**

**o Why is it a problem that women/menstruators are excluded from these studies? Explain the implications further.**

**o Do you want to add in the other side-effects the vaccine brings which have been documented widely? This allows you to point out the invisibility of menstruation/the disregard for women’s/menstruators health.**

**Author response:** Thank you for your comments. I appreciate your feedback, although I'm not entirely certain I understand your question. However, I have attempted to address your comment in the revised manuscript and have highlighted it according to my understanding. I hope there is no confusion, and the revisions adequately reflect your concerns.

**Comment 7: Conclusion**

**o create separate section for conclusion**

**o List again which symptoms of menstruation the vaccine affected**

**Author response:** Thank you for your suggestion. I have created a separate section for the conclusion in the revised manuscript to provide a clear summary of the findings and their implications. Additionally, I have listed the symptoms of menstruation that the vaccine affected again for emphasis and clarity.

**Thank you for your time and consideration.**

**Sincerely,**

**Warda Hassan Abdullah**
